# FDA approval of SetPoint System: the first neuroimmune modulation therapy for rheumatoid arthritis

**DOI:** 10.1097/MS9.0000000000004673

**Published:** 2026-01-06

**Authors:** Abdullah Imtiaz, Sakan Binte Imran, Ayesha Nazeer, Ehtisham Haider

**Affiliations:** a Department of Medicine, Wah Medical College, Wah Cantt, Rawalpindi, Pakistan; b Department of Medicine, Sir Salimullah Medical College, Dhaka, Bangladesh; c Department of Medicine, Punjab medical college, Faisalabad, Pakistan

*To the Editor,*

Rheumatoid arthritis (RA) is an autoimmune disorder characterized by joint pain, swelling, and long-term disability, affecting millions of people globally. Although there has been significant improvement in therapy, including biologic agents and targeted synthetic disease-modifying antirheumatic drugs (DMARDs), a large number of patients still experience partial responses, adverse effects, or treatment-related fatigue^[1,2]^. These limitations indicate the need for new, safer, and more specific treatment approaches. Previous studies, including those by Koopman *et al* and Bonaz, demonstrated that vagus nerve stimulation (VNS) can modulate inflammatory pathways in RA patients, but these were conducted under investigational device pathways and did not constitute regulatory approval^[3-5]^. The recent FDA premarket approval (PMA) of the SetPoint System represents the first regulatory authorization of an implanted neuroimmune-modulation therapy for adults with moderate-to-severe RA who are inadequate responders to, or intolerant of, biologic or targeted-synthetic DMARDs, defining a clear indication and regulatory pathway. This distinguishes the current approval from prior pilot studies and establishes the device as “first-in-class” in terms of regulatory status^[5]^.

The SetPoint System comprises a pulse generator (neurostimulator) and a lead with electrodes. The neurostimulator is implanted surgically below the left clavicle and attached to the left cervical vagus nerve through the lead^[6]^. The device delivers brief daily electrical pulses to the vagus nerve for 60 seconds, activating the cholinergic anti-inflammatory pathway, which modulates pro-inflammatory cytokine production and attenuates systemic inflammation^[7]^. The FDA approval was supported by the pivotal RESET-RA randomized, sham-controlled trial enrolling 242 patients. Publicly available summaries report that the primary endpoint, ACR20 at 3 months, was achieved by 35.2% of patients in the active-stimulation group versus 24.2% in the sham group^[8]^. Public summaries indicate clinical improvements beyond the 3-month time point, although detailed 12-month outcomes have not yet been published. Complete peer-reviewed results, including ACR50/70 responses, DAS28-CRP changes, effect sizes, and methods for handling missing data, remain unavailable. Device- or procedure-related serious adverse events were reported at 1.7%^[9]^, but detailed rates of implant-site infection, lead fracture, vocal-cord paresis, bradyarrhythmias, or battery-revision procedures in the RA cohort are not publicly available.

The introduction of the SetPoint System as the first FDA-approved bioelectronic medicine in RA represents a major breakthrough, providing a non-pharmacologic alternative for patients who are intolerant or non-responsive to conventional treatments. By targeting neural circuits that regulate immune function rather than broadly suppressing inflammation, it aims to restore immune balance while reducing dependence on lifelong pills, injections, and infusions^[4,5,7]^. Conventional RA therapies carry risks such as infections, malignancy, hepatotoxicity, bone-marrow suppression, and gastrointestinal intolerance, so contextualizing a non-pharmacologic approach is clinically relevant^[3]^. However, cost, reimbursement, and economic comparisons with long-term biologic therapy have not yet been publicly reported, limiting conclusions about real-world accessibility. While long-term effectiveness and extended safety remain to be determined, FDA approval marks a significant step forward in bioelectronic medicine and offers a promising new option for targeted control of chronic inflammatory disease. This manuscript complies with TITAN Guidelines, 2025, declaring no use of AI^[10]^.
